# Long-term outcomes after hospitalization for atrial fibrillation or flutter

**DOI:** 10.1093/eurheartj/ehae204

**Published:** 2024-04-28

**Authors:** Linh Thi Hai Ngo, Yang Peng, Russell Denman, Ian Yang, Isuru Ranasinghe

**Affiliations:** Faculty of Medicine, The University of Queensland, 627 Rode Road, Chermside, Queensland 4032, Australia; Department of Cardiology, The Prince Charles Hospital, 627 Rode Road, Chermside, Queensland 4032, Australia; Faculty of Medicine, The University of Queensland, 627 Rode Road, Chermside, Queensland 4032, Australia; Department of Cardiology, The Prince Charles Hospital, 627 Rode Road, Chermside, Queensland 4032, Australia; Department of Cardiology, The Prince Charles Hospital, 627 Rode Road, Chermside, Queensland 4032, Australia; Faculty of Medicine, The University of Queensland, 627 Rode Road, Chermside, Queensland 4032, Australia; Department of Thoracic Medicine, The Prince Charles Hospital, 627 Rode Road, Chermside, Queensland 4032, Australia; Faculty of Medicine, The University of Queensland, 627 Rode Road, Chermside, Queensland 4032, Australia; Department of Cardiology, The Prince Charles Hospital, 627 Rode Road, Chermside, Queensland 4032, Australia

**Keywords:** Atrial fibrillation, Hospitalization, Long-term, Outcomes

## Abstract

**Background and Aims:**

Atrial fibrillation (AF) and flutter are common causes of hospitalizations but contemporary long-term outcomes following these episodes are uncertain. This study assessed outcomes up to 10 years after an acute AF or flutter hospitalization.

**Methods:**

Patients hospitalized acutely with a primary diagnosis of AF or flutter from 2008–17 from all public and most private hospitals in Australia and New Zealand were included. Kaplan–Meier methods and flexible parametric survival modelling were used to estimate survival and loss in life expectancy, respectively. Competing risk model accounting for death was used when estimating incidence of non-fatal outcomes.

**Results:**

A total of 260 492 adults (mean age 70.5 ± 14.4 years, 49.6% female) were followed up for 1 068 009 person-years (PY), during which 69 167 died (incidence rate 6.5/100 PY) with 91.2% survival at 1 year, 72.7% at 5 years, and 55.2% at 10 years. Estimated loss in life expectancy was 2.6 years, or 16.8% of expected life expectancy. Re-hospitalizations for heart failure (2.9/100 PY), stroke (1.7/100 PY), and myocardial infarction (1.1/100 PY) were common with respective cumulative incidences of 16.8%, 11.0%, and 7.1% by 10 years. Re-hospitalization for AF or flutter occurred in 21.3% by 1 year, 35.3% by 5 years, and 41.2% by 10 years (11.6/100 PY). The cumulative incidence of patients undergoing catheter ablation of AF was 6.5% at 10 years (1.2/100 PY).

**Conclusions:**

Patients hospitalized for AF or flutter had high death rates with an average 2.6-year loss in life expectancy. Moreover, re-hospitalizations for AF or flutter and related outcomes such as heart failure and stroke were common with catheter ablation used infrequently for treatment, which warrant further actions.

## Structured Graphical Abstract

**Structured Graphical Abstract:**
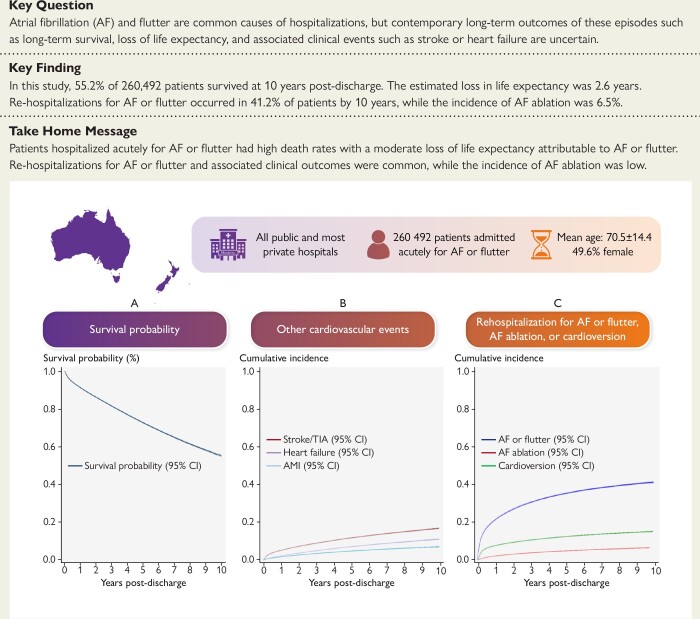
Long-term outcomes following a hospitalization for atrial fibrillation or flutter. AF, atrial fibrillation; AMI, acute myocardial infarction; CI, confidence interval; TIA, transient ischaemic attack

**See the editorial comment for this article ‘Atrial fibrillation and flutter: reiterating the need to ‘Go hard, Go early’, by A. Chauhan and A. Banerjee, https://doi.org/10.1093/eurheartj/ehae029.**

## Introduction

Atrial fibrillation (AF) and flutter are common causes of hospitalizations globally.^1–3^ Often occurring for symptomatic episodes of tachyarrhythmia, these hospitalizations are also costly, representing 50%–67% of AF-related health care expenditure.^4,5^ It is also well-established that AF and flutter are associated with an elevated risk of death, and clinical sequelae such as stroke, heart failure, and acute myocardial infarction.^6^ Encouragingly, early rates of death and readmission after AF hospitalizations have declined,^3^ a finding that could be attributed to advances in management such as the implementation of better integrated and structured care models incorporating multidisciplinary teams and community supports.^7^

In contrast, contemporary long-term outcomes of these episodes are poorly understood. Existing studies rarely report survival beyond 12 months with longer-term data limited to historical studies performed in the 1980s to 2000s.^8–10^ Furthermore, as these patients are usually elderly with multimorbidity, the loss in life expectancy attributable to AF or flutter compared with the general population is a more meaningful prognostic measure than survival in isolation, yet it has never been reported in a large-scale study. Moreover, long-term incidence of clinical sequelae such as stroke, heart failure, and acute myocardial infarction or the risk of re-hospitalizations for AF or flutter are uncertain with only limited data.^11–13^ Similarly, catheter ablation is advocated by contemporary guidelines to reduce the AF burden in symptomatic patients,^14^ yet the proportion of patients receiving catheter ablation downstream of an AF or flutter hospitalization is poorly understood. Evaluating these long-term outcomes is crucial for patients and clinicians seeking to better understand the prognosis of these chronic conditions. These outcomes are also a measure of success of therapeutic strategies such as anticoagulation for stroke prophylaxis and are essential to guide further clinical and policy efforts to improve care.

In this study, we examined clinical outcomes up to 10 years after an acute hospital admission for AF or flutter using national data from all public and most private hospitals in Australia and New Zealand from 2008–17. We specifically examined the long-term incidence of all-cause mortality and quantified the loss in life expectancy attributable to AF and flutter comparing with the general population. We also evaluated the longitudinal risk of associated cardiovascular outcomes such as stroke, heart failure, and acute myocardial infarction, as well as the need of re-hospitalization for AF or flutter, including for treatment with catheter ablation.

## Methods

### Data source

We used the Admitted Patient Collection of each state and territory in Australia and the equivalent National Minimum Dataset (Hospital Events) from New Zealand that record all hospital inpatient and day-only admissions at all public and most private hospitals. Data were not available from private hospitals in the Australian states of South Australia, Tasmania, and Northern Territory, whose collective population accounts for <10% of the total Australian population.^15^ Data from private hospitals in New Zealand were also not available. No changes in coverage occurred during the study period. For each encounter, a set of variables is recorded including the acute or elective status of the admission as defined by Australian Institute of Health and Welfare,^16^ demographic data, the primary diagnosis and up to 50 secondary diagnoses using International Classification of Diseases, 10th revision Australian Modification (ICD-10-AM) codes, up to 50 procedures performed using Australian Classification of Health Interventions (ACHI) codes, and patient’s status at discharge. Each encounter in Australia was linked to subsequent hospital admissions and Registry of Deaths by the designated Data Linkage Units in each state and territory with the accuracy of record linkage exceeding 99%.^17^ These linkages allowed us to identify re-hospitalizations to any hospitals and post-discharge deaths, including those occurring in-hospital and in community. In New Zealand, hospital encounters are linked nationally, and all deaths are recorded in the National Health Index Sociodemographic Profile. Prior studies have shown >85% accuracy for ICD-10-AM diagnosis and ACHI procedure coding compared with medical records^18^ with AF coding shown to have good (>85%) positive predictive value.^19^

### Study cohort

We included adults aged >18 years hospitalized between 2008 and 2017 inclusive, who were Australian or New Zealand residents and had AF or flutter (ICD-10-AM code I48 and all sub-codes) as the primary diagnosis. The study period 2008–17 was purposefully selected to allow robust estimate of long-term outcomes. The primary diagnosis is defined as the condition established after the admission to be chiefly responsible for occasioning the patient’s episode of care in the hospital. The primary diagnosis is determined by the clinical team and subsequently assigned an ICD-10-AM code by a trained coder according to the standardized Australian National Coding Standards. As the management of AF and flutter is fundamentally similar, and consistent with other studies,^2,3,20^ we included all hospitalizations for both conditions. We excluded patients who were discharged against medical advice. We only included acute (unplanned or emergency) admissions which are defined as encounters in which patients require emergency admission within 24 h.^16^ We excluded elective hospitalizations as these would typically represent admission for scheduled or planned rather than for acute care for AF or flutter. The acute/elective status for an admission is a mandatory field and completed for all patients. We have previously shown patients undergoing elective hospitalizations have a much lower risk of death or readmission compared with those acutely hospitalized, which is consistent with the findings that they are generally a more stable cohort undergoing planned or scheduled care.^21^ For patients that experienced multiple hospitalizations during the study period, only the first hospitalization was included (all subsequent hospitalizations were considered as an outcome of the index hospitalization).

### Outcomes

The primary outcome was all-cause death including those occurring in the community. Secondary outcomes were (i) loss in life expectancy attributable to AF or flutter; (ii) AF or flutter associated outcomes including stroke or transient ischaemic attack (TIA), heart failure, and acute myocardial infarction; (iii) re-hospitalizations for AF or flutter; and (iv) receipt of catheter ablation or cardioversion. Cause of re-hospitalization was identified based on the primary diagnosis coded for the re-hospitalization. Diagnosis and procedure codes used to identify catheter ablation of AF have been described in detail before^22–24^ and a full list of ICD-10-AM and ACHI codes used to define these outcomes are provided in Supplementary data online, *Table S1*.

### Statistical methods

Categorical data are presented as frequencies and percentages, while continuous variables are presented as mean ± standard deviation or as median and interquartile range (IQR). Differences between groups were tested by χ^2^ or Fisher’s exact test for categorical variables and Student’s *t*-test or Mann–Whitney *U* test for continuous variables where appropriate. We derived comorbidities using the Condition Category (CC) classification that groups ICD-10 diagnoses into 180 clinically meaningful conditions based on the selected secondary diagnoses of the index admission and all diagnoses codes from hospitalizations in the preceding 12 months.^25^

Survival probability was estimated using Kaplan–Meier time-to-event analysis and results reported as percentages and the corresponding 95% confidence intervals (CIs). We used a flexible parametric survival model to estimate the expected survival, observed survival, and the loss in life expectancy,^26^ adjusting for other patient characteristics and comorbidities that impacted survival. Variables for adjustment were identified by fitting a multivariable Cox regression model with mortality as the outcome variable. Independent variables included age, sex, presenting region, treatment at a private hospital, receipt of catheter ablation, cardioversion, or coronary angiography or percutaneous coronary intervention during the index hospitalization, and all comorbidities with a statistically significant relationship (*P* < .25) in a univariate analysis or clinically plausible relationship with the outcome of death. Expected life expectancy, matched for age, sex, and region, was estimated using life tables of the general population.^27,28^ Loss of life expectancy was calculated as the difference between the life expectancy of patients hospitalized for AF or flutter and the expected life expectancy estimated using the mortality data from the general population.^29^ When estimating the survival function for those with AF or flutter, the model uses the baseline survival function (i.e. with zero covariates) to ensure that the loss in life expectancy estimated is attributable to AF or flutter. Results are reported as the number of years lost as well as the proportion of life expectancy lost with the corresponding 95% CI. To calculate the incidence of non-fatal outcomes, we used Fine and Gray’s competing risk survival model with death being the competing event.^30^ Patients were censored if they did not experience the event at the end of follow-up or died before experiencing the outcome. The results were reported as incidence rate per 100 person-years (PY) and the overall cumulative incidence over the study period. The association between age group and sex with the risk of re-hospitalization for AF or flutter and the receipt of AF ablation, adjusted for other characteristics, was evaluated by using multivariable flexible parametric models with death being the competing event. Independent variables included patient demographic characteristics (age group, sex), presenting region, hospital sector, procedures performed during the index hospitalization (angiography or angioplasty, and direct cardioversion), and a range of cardiovascular and non-cardiovascular comorbidities. Final variables included in the models for death, AF or flutter re-hospitalizations, and AF ablation are presented in Supplementary data online, *Tables S2*, *S3*, and *S4*, respectively. All tests were two-sided and performed using Stata version 16.0, and a *P* < .05 was considered statistically significant.

### Role of the funding source

The funding organization had no role in the design, conduct, or reporting of this study.

## Results

### Study cohort

We identified 626 627 hospitalizations with a primary diagnosis of AF or flutter (see Supplementary data online, *Figure S1*). A total of 366 135 patients were excluded (not mutually exclusive) due to: not the first admission (264 783 hospitalizations, note that the first hospitalization of these patients was included in the cohort), elective (planned) hospitalization (217 757), and discharged against medical advice (2910). The final study cohort included 260 492 unique patients. The median follow-up time, estimated using the reverse Kaplan–Meier estimator,^31^ was 5.2 years (IQR 2.5–7.7 years). A total of 97 442, 53 450, and 17 436 of patients had a follow-up time of ≥5 years, ≥7 years, and ≥9 years, respectively.

### Patient characteristics

The mean age was 70.5 ± 14.4 years with most (69.1%) patients aged 65 or older (*Table 1*). The proportion of females and males were similar (49.6% vs. 50.4%). The median length of stay was 2 (IQR 1–5) days. The most common cardiac comorbidities were hypertension (22.4%), heart failure (17.7%), and coronary artery disease (11.7%). Diabetes (14.4%), chronic kidney disease (5.2%), and chronic lung disease (5.1%) were the most common non-cardiac comorbidities. The baseline characteristics of the study cohort by survival status, sex, and by two-year enrolment period are presented in Supplementary data online, *Tables S5*, *S6*, and *S7*, respectively.

**Table 1:** Cohort characteristics

	Overall (*N* = 260 492), *n* (%)
Patients’ demographics	
Age, years (mean ± SD)	70.5 ± 14.4
Age group, years, *n* (%)	
18–34	5211 (2.0)
35–49	18 307 (7.0)
50–64	57 040 (21.9)
65–79	102 322 (39.3)
≥80	77 612 (29.8)
Female sex, *n* (%)	129 295 (49.6)
Median length of stay, days (IQR)	2 (1–5)
Geographical region, *n* (%)	
New Zealand	41 615 (16.0)
Australian Capital Territory/New South Wales	77 661 (29.8)
South Australia/Northern Territory	16 820 (6.5)
Queensland	48 373 (18.6)
Tasmania	3922 (1.5)
Victoria	53 088 (20.4)
Western Australia	19 013 (7.3)
Treatment at a private hospital, *n* (%)	31 804 (12.2)
Procedures during the index hospitalization, *n* (%)	
Catheter ablation	860 (0.3)
Cardioversion	23 445 (9.0)
Coronary angiogram	7615 (2.9)
PCI	554 (0.2)
CHA_2_DS_2_-VASc score^a^	2 (1–3)
Cardiovascular history, *n* (%)	
Hypertension	58 430 (22.4)
Heart failure	46 073 (17.7)
Valvular and rheumatic heart disease	13 539 (5.2)
Coronary artery disease	30 587 (11.7)
Vascular disease	5860 (2.3)
History of AF or flutter^b^	34 608 (13.3)
Other comorbidities, *n* (%)	
Diabetes mellitus	37 536 (14.4)
Chronic lung diseases	13 312 (5.1)
Chronic kidney disease	13 503 (5.2)
Previous stroke or TIA	5143 (2.0)
Haematological disorders	19 230 (7.4)
Pneumonia	13 668 (5.3)
Musculo-skeletal and connective tissue disorders	24 781 (9.5)
Dementia and senility	6081 (2.3)
Major cancer	6965 (2.7)
End-stage liver disease	1047 (0.4)
Drug or alcohol abuse, psychosis, or dependence	9267 (3.6)
Psychiatric disorders	7338 (2.8)
Neurological disorders and paralysis	5206 (2.0)
Skin ulcers	2510 (1.0)
Urinary tract disorders and incontinence	15 407 (5.9)

SD, standard deviation; IQR, interquartile range; PCI, percutaneous coronary intervention; TIA, transient ischaemic attack.

^a^CHA_2_DS_2_-VASc score is a score used to evaluate risk of experiencing thrombo-embolic events of AF patients in which a point each is given for the presence of congestive heart failure (C), hypertension (H), age ≥ 65 years old (A), diabetes (D), vascular disease (VASc) and female gender and 2 points each are given for age ≥ 75 years old and history of stroke (S). The total score ranges from 0–9 with the higher the score, the higher the risk.^14^

^b^History of AF or flutter was derived from all acute and elective hospital encounters in the preceding 12 months.

### Study outcomes

#### Long-term survival

**Figure 1:**
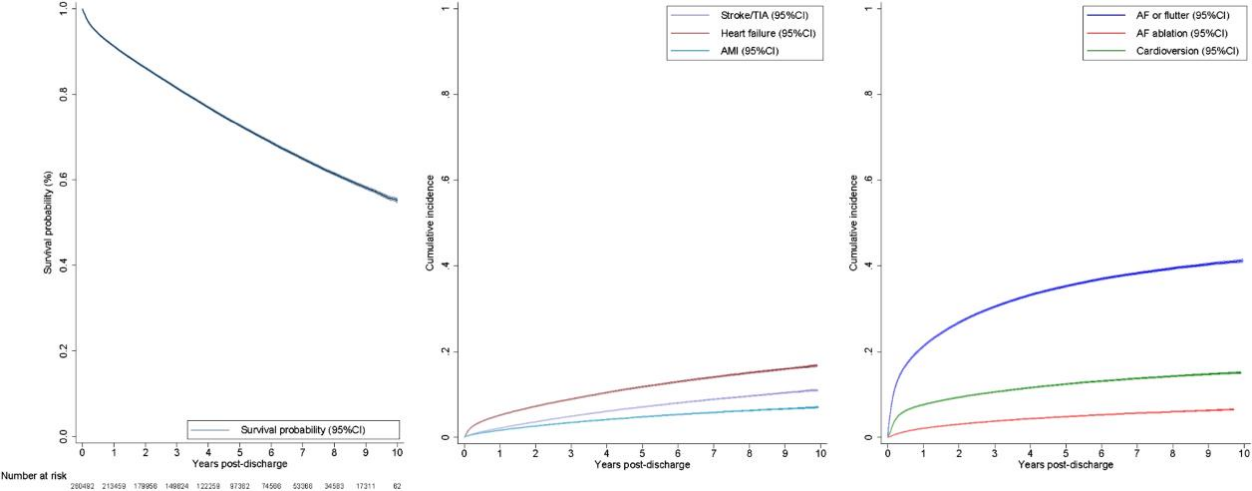
Long-term outcomes following a hospitalization for atrial fibrillation or flutter. (*A*) Cumulative incidence of mortality following a hospitalization for atrial fibrillation or flutter. (*B*) Cumulative incidence of hospitalizations for other cardiovascular events. (*C*) Cumulative incidence of re-hospitalizations for AF or flutter, catheter ablation of AF, and cardioversion. AF, atrial fibrillation; AMI, acute myocardial infarction; CI, confidence interval; TIA, transient ischaemic attack

A total of 69 167 patients died during 1 068 009 PY of follow-up (*Table 2* and *Figure 1*). At one year post-discharge, 91.2% (95% CI 91.1%–91.4%) of patients were surviving and declined to 72.7% (72.5%–72.9%) at 5 years and reached 55.2% (54.7%–55.7%) at 10 years. The incidence rate of death was 6.5/100 PY over the study period, which peaked in the first year post-discharge (9.4/100 PY) and gradually reduced to 5.7/100 PY at 1–5 years and 5.65/100 PY at 5–10 years. Observed survival was expectedly worse in the oldest age group (survival probability of 18.8% at 10 years) compared with younger age groups, and in females compared with males (see Supplementary data online, *Figure S2A*). After adjustment for differences in other characteristics, older age was expectedly associated with higher hazard of death compared with patients aged 18–34 years, while female sex was associated with lower hazard of death compared with males [hazard ratio (HR) 0.93, 95% CI 0.90–0.96, Supplementary data online, *Table S2*]. Survival was worse in those with comorbid heart failure and remained so after adjustment for other characteristics (see Supplementary data online, *Figure S3* and Supplementary data online, *Table S2*).

**Table 2:** Long-term outcomes of patients hospitalized acutely with atrial fibrillation or flutter from 2008–17

Outcomes	Total number of patients *N* (incidence rate)^a^	1-Year incidence rate *N* (incidence rate)	1–5-Year incidence rate *N* (incidence rate)	5–10-Year incidence rate *N* (incidence rate)	Cumulative incidence (95% CI)
Death	69 167 (6.5)	21 919 (9.4)	34 456 (5.7)	12 792 (5.6)	44.8% (44.3%–45.3%)
Re-hospitalizations for AF-associated outcomes					
Stroke/TIA	17 540 (1.7)	5431 (2.3)	9223 (1.6)	2885 (1.4)	11.0% (10.8%–11.2%)
Heart failure	29 194 (2.9)	12 987 (5.7)	12 505 (2.2)	3702 (1.8)	16.8% (16.6%–17.1%)
Acute myocardial infarction	11 720 (1.1)	4166 (1.8)	5897 (1.0)	1657 (0.8)	7.1% (6.9%–7.3%)
Re-hospitalizations for AF or flutter					
Any AF/flutter re-hospitalization	85 690 (11.6)	53 875 (27.2)	27 222 (6.6)	4593 (3.7)	41.2% (40.9%–41.6%)
Acute AF/flutter re-hospitalizations	64 982 (7.9)	36 333 (17.3)	24 462 (5.3)	4187 (2.9)	32.6% (32.3%–32.9%)
Catheter ablation or cardioversion					
Catheter ablation of AF	11 810 (1.2)	5299 (2.3)	5211 (0.9)	1300 (0.6)	6.5% (6.4%–6.7%)
Cardioversion	30 588 (3.2)	19 295 (8.7)	9207 (1.7)	2086 (1.1)	15.1% (14.9%–15.3%)

AF, atrial fibrillation; CI, confidence interval; TIA, transient ischaemic attack.

^a^Incidence rate per 100 person-years.

#### Loss in life expectancy attributable to AF or flutter

Compared with an age, sex, and region-matched general population, patients hospitalized for AF or flutter experienced, on average, a 2.6-year (95% CI 2.4–2.8 years) loss in life expectancy (*Table 3*). In proportional terms, this equated to a 16.8% (95% CI 15.7%–17.9%) reduction in the expected life expectancy. When different age groups were considered, the loss in life expectancy ranged from 1.6 years in those aged ≥80 years to 3.4 years in those aged 35–49 years. In proportional term, the highest loss (21.4%) was seen in those aged ≥80 years with the lowest (4.9%) seen in those aged 18–34 years. The years of life lost [2.7 years (95% CI 2.5–3.0) vs. 2.5 years (95% CI 2.3–2.6)] and proportion of life lost [16.4% (95% CI 15.3%–17.6%) vs. 17.2% (95% CI 16.1%–18.3%)] were comparable in males and females.

**Table 3:** Expected and observed life expectancy, loss in expectation of life, and proportion of expected life loss among patients hospitalized acutely for atrial fibrillation or flutter in Australia and New Zealand

Population	Expected life expectancy	Observed life expectancy (95% CI)	Loss in life expectancy (95% CI)	Proportion of life lost (95% CI)
Overall	18.0	15.4 (15.2–15.6)	2.6 (2.4–2.8)	16.8% (15.7%–17.9%)
Age				
18–34 years	46.6	44.3 (43.8–44.8)	2.3 (1.8–2.8)	4.9% (3.8%–5.9%)
35–49 years	39.2	35.8 (35.4–36.2)	3.4 (3.0–3.8)	8.7% (7.7%–9.6%)
50–64 years	27.1	23.8 (23.6–24.1)	3.3 (3.0–3.6)	12.1% (11.1%–13.0%)
65–79 years	15.7	12.8 (12.6–13.0)	2.9 (2.7–3.0)	18.0% (16.8%–19.1%)
≥80 years	7.3	5.7 (5.6–5.8)	1.6 (1.5–1.7)	21.4% (20.1%–22.7%)
Sex				
Female	16.2	13.7 (13.6–13.9)	2.5 (2.3–2.6)	17.2% (16.1%–18.3%)
Male	19.7	17.0 (16.7–17.2)	2.7 (2.5–3.0)	16.4% (15.3%–17.6%)

CI, confidence interval.

#### Re-hospitalization for AF-associated outcomes

A stroke or TIA occurred in 17 540 patients with a cumulative incidence of 11.0% (10.8%–11.2%) over the 10-year period. The overall incidence rate was 1.7/100 PY, which peaked in the first year at 2.3/100 PY, and then gradually declined to 1.4/100 PY at 5–10 years post-discharge. A total of 29 194 patients experienced at least one re-hospitalization for heart failure, with a cumulative incidence of 16.8% (95% CI 16.6%–17.1%), making it the most common AF-associated outcome. The overall incidence rate for heart failure re-hospitalizations was 2.9/100 PY with the rate highest in the first year (5.7/100 PY), reducing to 1.8/100 PY at 5–10 years post-discharge. Re-hospitalizations for acute myocardial infarction occurred infrequently with a cumulative incidence of 7.1% (95% CI 6.9%–7.2%) and an overall incidence rate of 1.1/100 PY. The incidence rate also declined gradually over time, from 1.8/100 PY in the first year to 0.8/100 PY at 5–10 years post-discharge. The incidences of re-hospitalization for stroke and TIA, heart failure, and acute myocardial infarction by sex and age group are presented in Supplementary data online, *Figure S4*.

#### Re-hospitalization for AF or flutter

The cumulative incidence of re-hospitalization for AF of flutter was 21.3% (95% CI 21.1%–21.4%) at 1 year, 35.3% (35.1%–35.5%) at 5 years, and 41.2% (40.9%–41.6%) by 10 years. The overall incidence rate of re-hospitalization for AF or flutter was 11.6/100 PY. The incidence rate peaked in the first year after discharge (27.2/100 PY), reducing to 6.6/100 PY at 1–5 years and 3.7/100 PY at 5–10 years post-discharge. The cumulative incidence of re-hospitalization for AF or flutter was lowest in patients aged ≥80 years and highest in patients aged 50–64 years and was comparable in males and females (see Supplementary data online, *Figure S2B*). After adjustment, risk of re-hospitalization for AF or flutter was higher among older age groups when compared with those aged 18–34 years, while sex had no significant relationship (see Supplementary data online, *Table S3*). The cumulative incidence was lower in those with comorbid heart failure and remained marginally lower after adjustment for other covariates (see Supplementary data online, *Figure S3B* and Supplementary data online, *Table S3*).

Among 85 690 patients with at least one re-hospitalization for AF or flutter, 64 982 or 76% occurred as an acute presentation to hospital with an overall incidence rate of 7.9/100 PY and cumulative incidence of 32.6% (95% CI 32.3%–32.9%).

#### Receipt of catheter ablation or cardioversion for AF

During the index hospitalization, 860 (0.3%) patients underwent catheter ablation for AF. Following discharge, AF ablation was utilized in 11 810 patients, at an incidence rate of 1.2/100 PY with a cumulative incidence of 6.5% (95% CI 6.4%–6.7%) by 10 years. The use of catheter ablation peaked in the first year (2.3/100 PY) and significantly declined in the 5–10-year period (0.6/100 PY) after discharge. Out of the 11 810 patients that underwent ablation, 9087 and 4951 had one and two acute re-hospitalizations for AF or flutter, respectively, before receiving AF ablation. Catheter ablation of AF was performed more frequently in those aged 35–64 years and in men (see Supplementary data online, *Figure S2C*). Following adjustment for other variables, age ≤ 65 years was associated with higher likelihood of receiving AF ablation compared with those aged 18–34 years, while female sex was associated with lower likelihood compared with males (HR 0.85, 95% CI 0.81–0.88, *P* < .001; see Supplementary data online, *Table S4* for more details). The cumulative incidence of ablation was lower in those with comorbid heart failure and remained lower after adjustment for covariates (see Supplementary data online, *Figure S3C* and Supplementary data online, *Table S4*). During the index hospitalization, 23 455 (9.0%) patients underwent cardioversion. Following discharge, cardioversion was performed in 30 588 patients with an incidence rate of 3.2/100 PY and a cumulative incidence of 15.1% (95% CI 14.9%–15.3%) by 10 years.

## Discussion

In this large contemporary cohort of patients hospitalized for AF or flutter, we found a survival of 55.2% at 10 years after hospitalization and a 2.6-year loss in life expectancy, or 16.8% of their expected life expectancy, attributable to AF or flutter compared with the general population. Cumulative incidence of AF and flutter associated outcomes such as stroke or TIA, heart failure, and acute myocardial infarction were relatively common with 1 in 10 patients experiencing a stroke and 1 in 6 re-hospitalized for heart failure. Re-hospitalization for AF or flutter was also exceedingly common, occurring in two out of five patients in the decade after the initial admission. Despite such high burden of AF re-hospitalization, <7% of patients underwent catheter ablation of AF during the follow-up period, suggesting potential under-utilization of this procedure in this symptomatic population (*Structured Graphical Abstract*). Collectively, these observations suggest that more effective strategies are urgently needed to reduce the AF burden and prevent downstream adverse cardiovascular events.

This study provides much-needed data on contemporary long-term outcomes for a rapidly growing cohort of patients hospitalized for AF of flutter. While several studies report the risk of death up to 1 year,^1–3^ to our knowledge, there are no large-scale population studies that provide unbiased estimates of survival beyond this period with existing data limited to historical studies performed in the 1980s–2000s.^8–10^ Encouragingly, we observed substantially better 10-year survival (55.2%) compared to these historical studies (30%–34% at 10 years),^8–10^ which is likely attributable to advances in AF and broader medical care^7^ even though our study cohort was slightly younger (mean age of 70.5 vs. 73–76 years old) and had lower rate of comorbidities.^8,9^ We extend the literature by undertaking a robust population-wide estimate of loss in life expectancy attributable to AF or flutter for the first time, a measure that is crucial to understand survival given death may be common in this cohort due to older age and the coexistence of AF and flutter with a myriad of comorbidities. We found that AF or flutter hospitalizations have an appreciable and meaningful detrimental impact on prognosis even in older adults given that these patients experienced a loss of 2.6 years in life expectancy, or nearly one-fifth of their anticipated life in proportional terms, compared with the general population.

We also found long-term clinical outcomes associated with AF and flutter to be relatively common. Knowledge of these risks is often sought by patients and physicians and crucial for disease prevention strategies yet rarely reported in the literature. Indeed, the only recent data on stroke and acute myocardial infarction risk stem from a sample representing 2.2% of the South Korean population, which showed a 9.6% cumulative incidence of stroke or TIA at 7 years^11^ and a 2.5% incidence of myocardial infarction at 5 years.^12^ Similarly, there are no large-scale studies outlining the long-term risk of heart failure with a few studies reporting conflicting incidence in AF patients at 2–3 years.^13,32^ We show that these AF-associated outcomes are common in the long-term, with an acute stroke or TIA occurring in one out of 10 patients, and heart failure severe enough to warrant hospitalization occurring in one in six by 10 years, emphasizing the critical need to implement more effective strategies to prevent these adverse clinical events. Global evidence has consistently shown that oral anticoagulation use, adherence, and persistence for the prevention of thrombo-embolic stroke is suboptimal.^33,34^ For example, in the National Cardiovascular Data Registry’s Practice Innovation and Clinical Excellence (PINNACLE) AF Registry, anticoagulation prescription did not exceed 50% even in those at high risk of stroke, highlighting significant opportunities to reduce the risk of embolic stroke by optimizing their use.^35^ Early rhythm control represents another promising solution with emerging evidence showing a 21% reduction in death and adverse cardiovascular events, including stroke, heart failure, and myocardial infarction by 5 years.^36^ Wider implementation of these evidence-based strategies offers significant opportunities to further reduce the risk of adverse cardiovascular events associated with AF and flutter.

Another novel finding in our study is re-hospitalization for AF or flutter was exceedingly common with one or more re-hospitalization occurring in two out of every five patients by 10 years, representing a significant burden to patients and the health care system that requires urgent action. Indeed, the risk of re-hospitalization for AF or flutter remains elevated well beyond the 1 year post-discharge period that most previous studies focused on. Despite this high AF re-hospitalization burden, <7% of patient underwent AF ablation during the index hospitalization or in the follow-up period. Prior study of claims data from the USA suggests that ablation is often considered second or third line treatment with only 5.2% of patients eventually having the procedure.^37^ Similarly, in the PINNCALE AF registry, only 2.9% of >500 000 AF patients underwent ablation.^38^ Our findings, in the context of these prior studies,^37,38^ suggesting that utilization of AF ablation remains low. We can only hypothesize that the low use may reflect perceived invasiveness of ablation and concerns about safety despite improvements in procedural complications over time,^23^ lack of awareness of long-term benefits,^39^ or the disparities in access to AF ablation observed in our data and those of others. For example, females accounted for half of all hospitalized AF patients yet only constituted 30% of all ablations in Australia and New Zealand during the same period.^22^ Furthermore, we found nearly 90% of acute AF hospitalizations occurred in public hospitals, yet more than 75% of all AF ablations were performed in private hospitals,^24^ implying substantial financial barriers to accessing ablation. Reducing these disparities, improving access to ablation, and implementation of other proven strategies such as weight loss, alcohol abstinence, and better management of risk factors such as hypertension^40^ may reduce the AF burden and minimize re-hospitalizations.

There are limitations that should be considered when interpreting our results. Routinely collected hospitalization data are generally considered less granular than those collected specifically for research purposes^41^ which may influence the estimates in our study. Nevertheless, studies have shown good accuracy of coded data when compared with medical records^18^ that is only modestly lower than that of clinical registries^41^ with high accuracy for identifying AF.^19^ Data from some private hospitals were not available to researchers, even though most acute AF or flutter patients were treated in public hospitals. Variables that may impact long-term outcomes such as the type of AF, patient vital signs, investigation results, and medications are not recorded in administrative datasets and were not accounted for in our analysis. Specifically, our study could not account for potential differences in the management of AF and atrial flutter or among different subtypes of AF. Furthermore, prescription of oral anticoagulant (OAC) including the uptake of novel oral anticoagulants was not available and may influence the outcomes reported in this study. Nevertheless, global^42–44^ and local^45,46^ literature has shown that even though OAC use has increased by varying degrees (8% increase in the USA,^43^ up to 20% change in Germany,^44^ and 12.5% in Australia^45^), its use remains suboptimal.^42,45,46^ Whether treatments provided were adherent with guidelines recommendations for any individual patient was not captured in administrative data. Thus, the effect of appropriateness of treatment decisions on outcomes could not be evaluated. We report average outcomes over then 10-year study period, and it is possible that treatments for AF and flutter have changed over the period that may have influenced outcomes. We evaluated patients hospitalized for AF or flutter and therefore, our findings may not be applicable to patients solely diagnosed and managed in the community. Clinical events that did not require hospitalization were also not captured. Nevertheless, re-hospitalizations are likely to reflect the clinically significant events which have the most impact on patient prognosis and the health care system. Lastly, some patients may have permanently emigrated from Australia and New Zealand and were lost to follow-up. However, permanent emigration is uncommon (0.3%–0.4% of the population in Australia per annum^47^), thus lost to follow-up is likely to be small and unlikely to markedly change the average re-hospitalization and survival values reported.

## Conclusions

Patients hospitalized for AF and flutter had a low survival of 55.2% at 10 years with an attributable loss of life expectancy of 2.6 years or nearly one-fifth of their anticipated life expectancy. AF-associated cardiovascular outcomes were relatively common, and so was re-hospitalization for AF or flutter, which occurred in two out of every five patients by 10 years. Despite the high burden of AF or flutter re-hospitalizations, the incidence of AF ablation was low with <7% of patients receiving this procedure at 10 years post-discharge. Effective strategies are needed to reduce the AF burden and decrease the downstream risk of AF and flutter-related adverse cardiovascular events.

## Supplementary Material

ehae204_Supplementary_Data

## Data Availability

The data underlying this article were provided by a third party including the Data Custodian Units of each state and territory in Australia under ethics approval and New Zealand Ministry of Health via a data user agreement. Data could be accessed upon request to the third party.

## Pre-registered Clinical Trial Number

None supplied.
