# Supplementary material for: Long-term outcomes after hospitalization for atrial fibrillation or flutter
Source: Eur Heart J. 2024 Apr 28;45(24):2133–41. doi: 10.1093/eurheartj/ehae204 (PMC11212827; doi:10.1093/eurheartj/ehae204)
Supplement: ehae204_Supplementary_Data [file ehae204_supplementary_data.docx]

**Supplemental Appendix**

**Longitudinal Outcomes Following Hospitalisation for Atrial Fibrillation or Flutter**

Linh Ngo, Yang Peng, Russell Denman, Ian Yang, Isuru Ranasinghe

**Supplemental Figure**

**Figure S1:** Patient selection flow diagram

**Figure S2:** Long-term outcomes following a hospitalisation for AF or flutter by age group and sex. A) Long-term mortality; B) Incidence of re-hospitalisations for AF or flutter; C) Incidence of catheter ablation of AF. Abbreviations: AF=atrial fibrillation, HF=heart failure.

**Figure S3:** Long-term outcomes following a hospitalisation for AF or flutter by comorbid heart failure. A) Long-term mortality; B) Incidence of re-hospitalisations for AF or flutter; C) Incidence of catheter ablation of AF.

**Figure S4:** Long-term AF related outcomes following a hospitalisation for AF or flutter by age group and sex. A) Incidence of Stroke and TIA; B) Incidence of heart failure; C) Incidence of AMI. Abbreviations: AMI = acute myocardial infarction, TIA = transient ischaemic attack.

**Supplemental Tables**

**Table S1:** Diagnosis and procedure codes used in this study

**Table S2:** Variables associated with long-term mortality in the flexible parametric model

**Table S3**: Variables associated with the outcome of re-hospitalisation for atrial fibrillation or flutter

**Table S4**: Variables associated with the outcome of re-hospitalisation for catheter ablation of atrial fibrillation

**Table S5:** Baseline characteristics by survival status at the end of the study period

**Table S6:** Baseline characteristics by sex

**Table S7:** Baseline characteristics by two-year enrolment period

**Figure S1: Patient selection flow diagram**


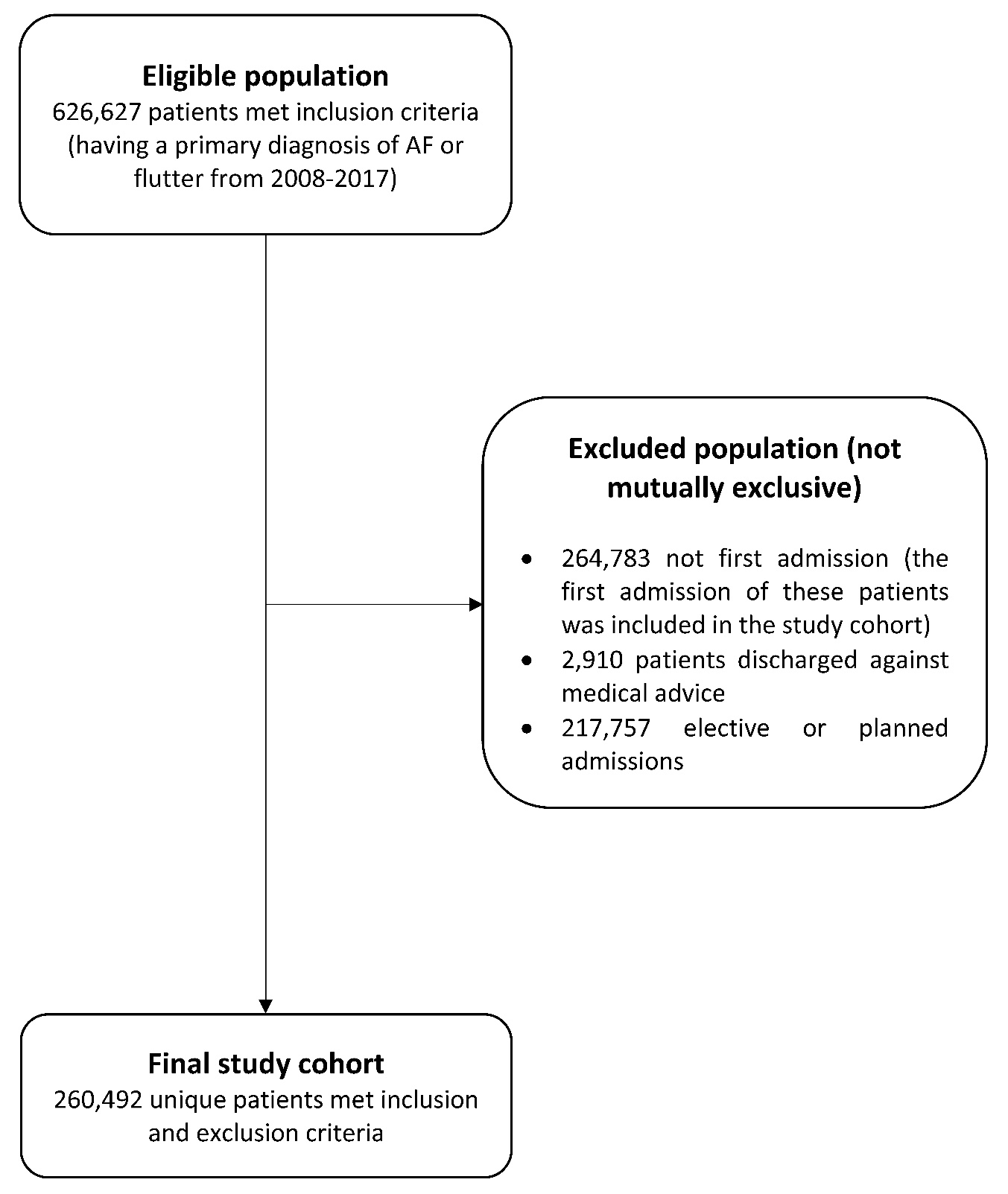


**Figure S2: Long-term outcomes following a hospitalisation for AF or flutter by age group and sex. A) Long-term mortality; B) Incidence of re-hospitalisations for AF or flutter; C) Incidence of catheter ablation of AF. Abbreviations: AF=atrial fibrillation, HF=heart failure.**

**
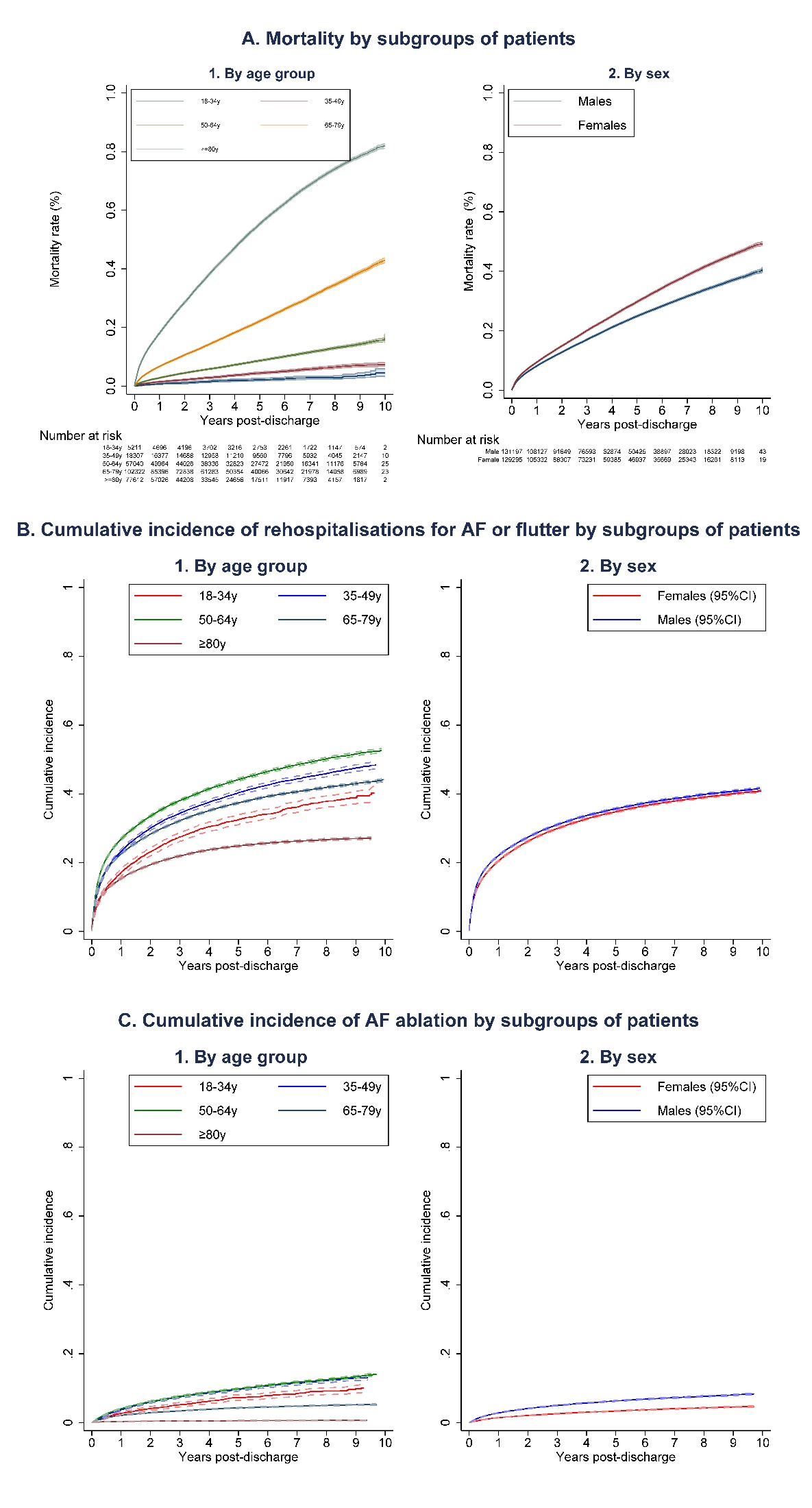
**

**Figure S3: Long-term outcomes following a hospitalisation for AF or flutter by comorbid heart failure.**


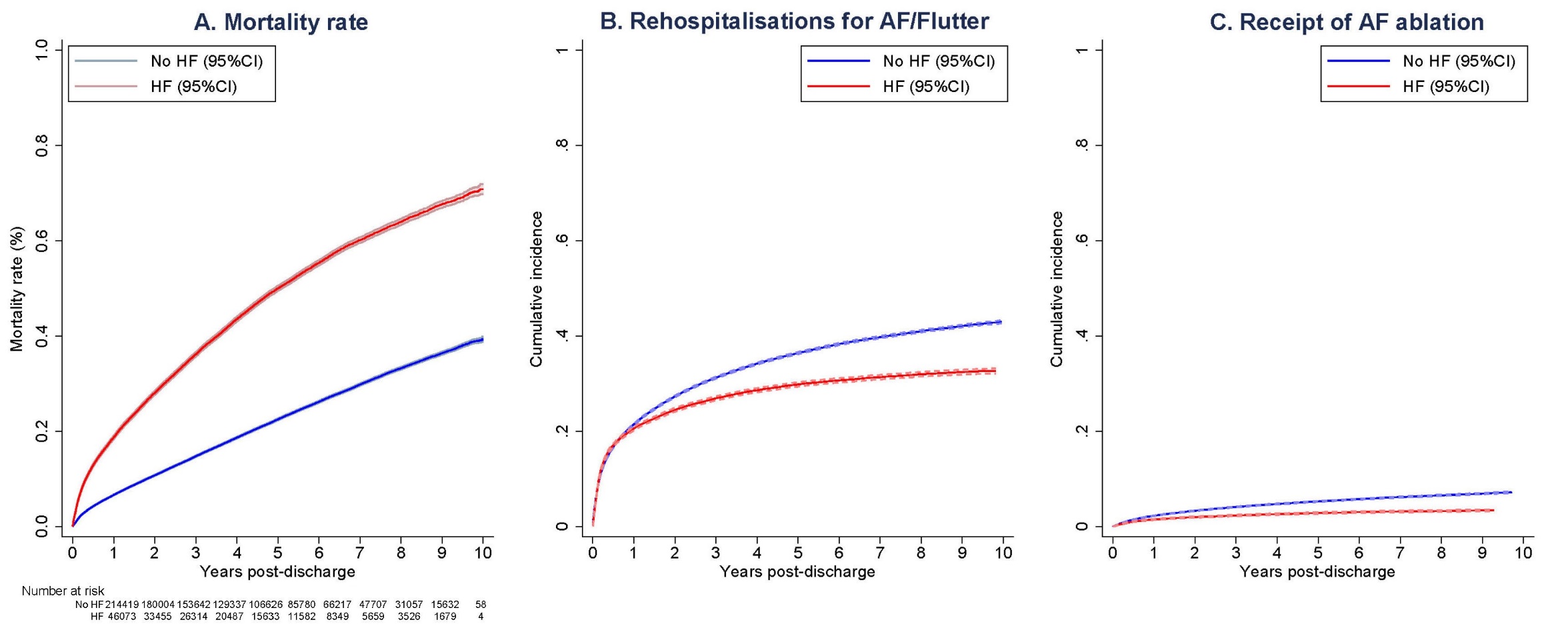


**Figure S4: Long-term AF-related outcomes following a hospitalisation for AF or flutter by age group and sex. A) Incidence of Stroke and TIA; B) Incidence of heart failure; C) Incidence of AMI. Abbreviation: AMI = acute myocardial infarction, TIA = transient ischaemic attack.**


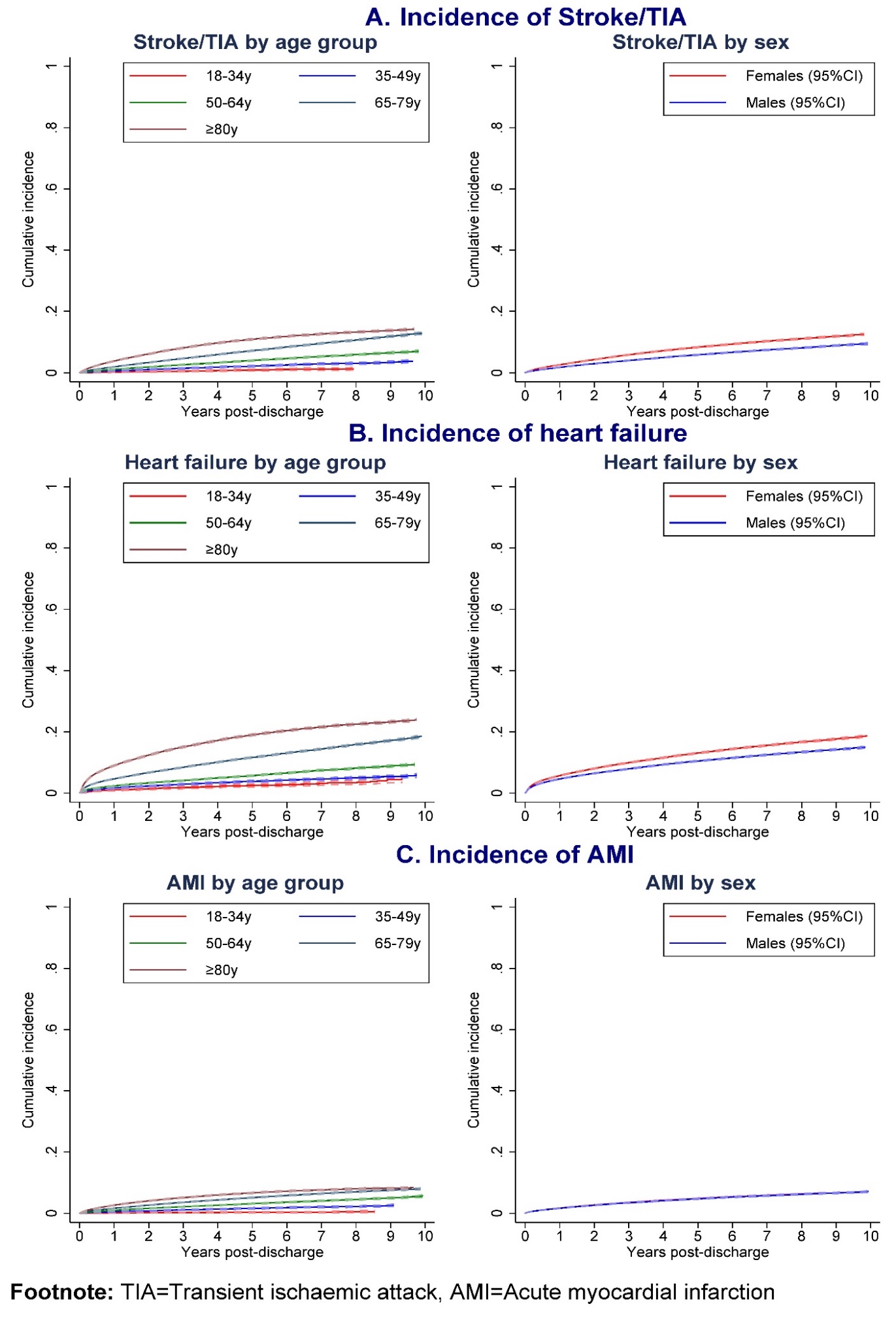


**Table S1: Diagnosis and procedure codes used in this study**

| **DISEASE/PROCEDURE** | **ICD10-AM/ACHI codes** |
| --- | --- |
| Atrial fibrillation | I48, I48.0, I48.1, I48.2, I48.9 |
| Atrial flutter | I48.3, I48.4 |
| Pre-excitation syndrome | I45.6 |
| Supra-ventricular tachycardia | I47.1 |
| Ventricular tachycardia | I47.2, I49.0 |
| Premature beats | I49.1, I49.2, I49.3, I49.4 |
| Other arrhythmias | I44, I47, I47.0, I49.1, I49.8, I49.9, R00.0 |
| Presence of a cardiac device | Z95.0 |
| Stroke | I60*, I61*, I62*, I63*, I64* |
| Transient ischemic attack | G45* |
| Heart failure | I110, I130, I132, I50, I500, I509, U822 |
| Acute myocardial infarction | I21* |
| Catheter ablation | 38287-01, 38287-02, 38290-01 |
| Pacemaker implantation | 38353-00 |
| Cardiac defibrillator implantation | 38393-00 |
| Open ablation | 38287-03, 38287-04, 38290-02 |
| Hypertension | I13*, I12*, I67.4, I11*, I10, I15* |
| Valvular and rheumatic heart disease | I01*, Q25.1, Q25.4, Q25.2, Q25.3, I35*, Q23.1, Q23.0, Q23.2, Q23.3, Q22.1, Q22.2, Q22.3, Q22.4, Q22.5, I39.0, I39.1, I39.2, I39.3, I39.4, I05*, I34*, I08*, I36*, I37*, I06*, I02.0, I00, I09*, I07* |
| Coronary artery disease | I21*, I22*, I23*, I51.1, I51.2, I24*, I20*, I25*, Q245 |
| Vascular disease | A48.0, I26*, I70*, I71*, I72*, I73*, I74*, I77*, I78.0, I79*, I80.1, I80.2, I82*, K55*, N28.0, N39.81, R02 |
| Diabetes mellitus | E09*, E10*, E11*, E12*, E13*, E14*, G453, H34*, H35.0, H35.1, H35.2, H35.6, H36.0, H36.8, H43.1, H45.0, Z48.0, Z51.81 |
| Chronic lung diseases | D86*, J41*, J42, J43*, J44*, J47, J60, J61, J62.0, J62.8, J63*, J64, J65, J66*, J67*, J68*, J70*, J82, J84*, J98.2, J98.3, J99* |
| Chronic kidney disease | Z49*, Z99.2, T85.71, N17*, N18*, I13.1, I13.2, I12.0, Q61.1, Q62*, N19 |
| Previous stroke or TIA | I61*, I62* I60*, I63*, G463, G464, I64, G45*, I66*, I65*, G46* |
| Haematological disorders | D45, D50*, D51*, D52*, D53*, D55*, D56*, D57*, D58*, D59*, D60*, D61*, D62, D63*, D64*, D65, D66, D67, D68*, D69*, D70, D71, D72*, D73*, D74*, D75*, D77, D80*, D81*, D82*, D83*, D84*, D89.2, D89.8, D89.9, I88*, R72 |
| Pneumonia | A06.5, A20.2, A21.2, A22.1, A42.0, A43.0, A48.1, A48.2, B01.2, B05.2, B38*, B39*, B66.4, B67.1, J10.0, J11.0, J12*, J13, J14, J15*, J16.0, J16.8, J17*, J18*, J69*, J85*, J86.0, J86.9, J92.0, J92.9, J94.1, R09.1 |
| Musculo-skeletal and connective tissue disorders | A54..4, A66.6, E55.0, H19.3, L40.5, L87.1, L94.4, M00*, M01*, M02*, M03*, M05*, M06*, M07*, M08*, M09*, M10*, M11*, M12*, M13*, M14*, M15*, 16*, M17*, M18*, M19*, M20*, M21*, M22*, M23*, M24*, M25*, M30*, M31*, M32*, M33*, M34*, M35*, M36*, M40*, M41*, M42*, M43*, M45*, M46*, M47*, M48*, M49*, M50*, M51*, M51*, M53*, M54*, M60*, M61*, M62*, M63*, M65*, M66*, M67*, M68*, M70*, M71*, M72*, M73*, M75*, M76*, M77*, M79*, M80*, M81*, M82*, M83*, M84*, M85*, M86*, M87*, M88*, M89*, M90*, M91*, M92*, M93*, M94*, M95*, M96*, Q35*, Q36*, Q37*, Q65*, Q66*, Q67*, Q68*, Q69*, Q70*, Q71*, Q72*, Q73*, Q74*, Q76*, Q77*, Q78*, Q79*, R25.2, R26.2, R29.4, S13*, S23*, S33*, Z45*, Z46* |
| Dementia and senility | A81*, E75*, F00*, F01*, F02*, F03, F04, F05.1, F06.8, F06.9, F70*, F09, F84.2, G13.2, G13.8, G30*, G31*, G32.8, G91*, G93*, G94*, G96*, G97.0, G98, G99.8, I67.3, R54 |
| Major cancer | C01, C02*, C03*, C04*, C05*, C06*, C07, C08*, C09*, C10*, C11*, C12, C13*, C14*, C15*, C16*, C17*, C22*, C23, C24*, C25*, C30*, C31*, C32*, C33, C34*, C37, C38*, C39*, C40*, C41*, C45*, C46*, C47*, C48*, C49*, C56, C57*, C58, C70*, C71*, C72*, C74*, C75*, C77*, C78*, C79*, C81*, C82*, C83*, C84*, C85*, C86*, C88*, CC90*, C91*, C92*, C93*, C94*, C95*, C96*, C97 |
| End-stage liver disease | I98.21, I98.2, I98.20, I85.9, I85.0, K76.7, K72.1, K72.9, K76.6, K70*, K74*, K71.7 |
| Drug or alcohol abuse, psychosis or dependence | F10*, F11*, F12*, F13*, F14*, F15*, F16*, F17*, F18*, F19*, F55* |
| Psychiatric disorders | F20*, F21, F22*, F23*, F24, F25*, F28, F29, F30*, F31*, F32*, F33*, F34*, F388*, F39, F40*, F41*, F42*, F43*, F44*, F45*, F48*, F50*, F51*, F52*, F54, F59, F60*, F61, F62*, F63*, F64*, F65*, F66*, F68*, F69, F91*, F92*, F94.0, F95*, F98*, F99, R45* |
| Neurological disorders and paralysis | G931 G936 E035 R402 G935 |
| Skin ulcers | L97, L97.9, L89*, L984 |
| Urinary tract disorders and incontinence | N02*, N06*, N10, N11*, N12, N13*, N15.1, N20*, N21*, N22.0, N22.8, N25*, N26, N27*, N28*, N29.1, N30*, N31*, N32*, N34*, N35*, N36*, N37.0, N37.8, N39*, Q60*, Q61*, Q62*, Q63*, Q64*, R31, R33, T19*, Z90.5 |
| Septicaemia or shock | A22.7, A39*, A40*, A41*, A41.8, A41.9, B00.7, P36*, P39*, R57.1, R57.8 |
| Protein-calorie malnutrition | E40, E41, E42, E43, E44.0, E44.1, E45, E46, E64.0, R09.0, R26.8, R29.81, R45*, R46*, R64 |
| Disorders of fluid or electrolyte acid-base balance | E86, E87* |
| Gallbladder and biliary tract disorders | K80*, K81*, K82*, K83*, K87.0, K91.5 |
| Intestinal obstruction or perforation | K25*, K26*, K27*, K28*, K56*, K63.1, K65*, K67* |
| Peptic Ulcer, haemorrhage, other specified gastrointestinal disorders | A02*, A03*, A04*, K22.1, K22.6, K25*, K26*, K27*, K28*, K29.0, K31*, K38.2, K41*, K42*, K43*, K44*, K45*, K46*, K55.21, K55.22, K57*, K63*, K66*, K92*, K93.8 |
| Delirium and encephalopathy | F05.0, F05.8, F05.9, F06*, G13.1, G93.3, G93.4, R41*, R44*, R52*, R53, R68.0, R68.8 |
| Mononeuropathy, other neurological conditions or injuries | B02.0, B02.2, G23.1, G24*, G25*, G26, G43*, G44*, G50*, G51*, G52*, G53*, G54*, G55*, G56*, G57*, G58*, G59.0, G59.8, R25*, R29*, S04*, S14*, S24*, S34*, S44*, S54*, S64*, S74*, S84*, S94*, T06.2, T09.4, T11.3, T13.3, T14.4 |
| Cardiorespiratory failure and shock | I46*, J80, J81, J95*, J96*, R45.7, R57.0, R57.9, R96.0, R96.1, R98, R99 |
| Asthma | J45*, J46 |
| Pleural effusion or pneumothorax | J90, J91, J93*, J94* |
| Cataract | H25*, H26*, H28* |
| Incontinence | N39.4, R15, R32, R36 |
| Cellulitis, local skin infection | A06.7, A20.1, A22.0, A31.1, A36.3, A43.1, A46, L03*, L04*, L08*, L88, L98.0, L98.3 |

Footnote: ACHI = Australian Classification of Health Interventions; ICD10-AM = International Classification of Diseases, 10^th^ Revision, Australian Modification. *All individual codes are included.

**Table S2:** **Variables associated with long-term mortality in the flexible parametric model**

| **Variables** | **HR** | **95%CI** | **P value** |
| --- | --- | --- | --- |
| **Presenting region** |  |  |  |
| Australian Capital Territory/New South Wales | Reference |  |  |
| Northern Territory/South Australia | 1.04 | 0.97 – 0.11 | 0.278 |
| New Zealand | 1.42 | 1.36 – 1.48 | <0.001 |
| Queensland | 0.97 | 0.92 – 1.01 | 0.131 |
| Tasmania | 1.27 | 1.13 – 1.42 | <0.001 |
| Victoria | 0.78 | 0.75 – 0.82 | <0.001 |
| Western Australia | 1.48 | 1.41 – 1.56 | <0.001 |
|  |  |  |  |
| **Age group** |  |  |  |
| 18-34 years | Reference |  |  |
| 35-49 years | 1.70 | 1.36 – 2.13 | <0.001 |
| 50-64 years | 2.59 | 2.10 – 2.30 | <0.001 |
| 65-79 years | 4.40 | 3.57 – 5.44 | <0.001 |
| ≥80 years | 7.52 | 6.09 – 9.29 | <0.001 |
|  |  |  |  |
| **Sex** |  |  |  |
| Male | Reference |  |  |
| Female | 0.93 | 0.90 – 0.96 | <0.001 |
|  |  |  |  |
| **Hospital sector** |  |  |  |
| Public hospital | Reference |  |  |
| Private hospital | 0.73 | 0.69 – 0.77 | <0.001 |
|  |  |  |  |
| **Procedures performed during the index hospitalisation** |  |  |  |
| Catheter ablation of AF | 0.47 | 0.32 – 0.69 | <0.001 |
| Coronary angiogram/PCI | 0.57 | 0.50 – 0.64 | <0.001 |
| Direct cardioversion | 0.62 | 0.57 – 0.66 | <0.001 |
|  |  |  |  |
| **Cardiovascular comorbidities** |  |  |  |
| Hypertension | 1.00 | 0.96 – 1.04 | 0.893 |
| Congestive heart failure | 2.68 | 2.59 – 2.76 | <0.001 |
| Valvular heart disease | 0.88 | 0.82 – 0.94 | <0.001 |
| Coronary artery disease | 1.01 | 0.96 – 1.05 | 0.783 |
| Vascular disease | 1.08 | 1.02 – 1.15 | 0.008 |
| History of hospitalisation with AF or flutter | 0.99 | 0.96 – 1.03 | 0.663 |
|  |  |  |  |
|  |  |  |  |
| **Non-cardiovascular comorbidities** |  |  |  |
| Diabetes | 1.46 | 1.41 – 1.52 | <0.001 |
| Chronic lung diseases | 1.87 | 1.78 – 1.96 | <0.001 |
| Chronic kidney disease | 1.52 | 1.46 – 1.60 | <0.001 |
| History of stroke | 1.03 | 0.95 – 1.11 | 0.485 |
| Haematological disorders | 1.17 | 1.12 – 1.23 | <0.001 |
| History of pneumonia | 1.22 | 1.16 – 1.28 | <0.001 |
| Musculo-skeletal and connective tissue disorders | 1.11 | 1.07 – 1.16 | <0.001 |
| Major cancer | 7.03 | 6.72 – 7.36 | <0.001 |
| Other cancers | 1.18 | 1.13 – 1.24 | <0.001 |
| Dementia | 2.24 | 2.08 – 2.40 | <0.001 |
| Drug and alcohol-related disorders | 1.39 | 1.30 – 1.49 | <0.001 |
| Mental | 1.12 | 1.05 – 1.20 | 0.001 |
| Skin ulcers | 1.44 | 1.34 – 1.54 | <0.001 |
| Urinary tract infection and incontinence | 1.18 | 1.13 – 1.23 | <0.001 |
| Neurological disorders and paralysis | 1.33 | 1.24 – 1.42 | <0.001 |
| Septicaemia or shock | 0.96 | 0.90 – 1.04 | 0.337 |
| Protein-calorie malnutrition | 1.27 | 1.20 – 1.34 | <0.001 |
| Disorders of fluid or electrolyte acid-base balance | 1.19 | 1.14 – 1.24 | <0.001 |
| Gallbladder and biliary tract disorders | 1.20 | 1.08 – 1.34 | 0.001 |
| Intestinal obstruction or perforation | 1.02 | 0.92 – 1.14 | 0.653 |
| Peptic Ulcer, haemorrhage, other specified gastrointestinal disorders | 0.95 | 0.89 – 1.01 | 0.074 |
| Delirium and encephalopathy | 1.18 | 1.12 – 1.25 | <0.001 |
| Mononeuropathy, other neurological conditions or injuries | 0.89 | 0.81 – 0.98 | 0.017 |
| Cardiorespiratory failure and shock | 0.96 | 0.89 – 1.03 | 0.248 |
| Asthma | 0.80 | 0.69 – 0.93 | 0.004 |
| Pleural effusion or pneumothorax | 1.18 | 1.10 – 1.27 | <0.001 |
| Cataract | 1.05 | 0.98 – 1.13 | 0.185 |
| Cellulitis, local skin infection | 1.20 | 1.12 – 1.28 | <0.001 |

Footnote: AF=atrial fibrillation, CI=confidence interval, HR=hazard ratio, PCI=percutaneous coronary intervention.

**Table S3**: **Variables associated with the outcome of re-hospitalisation for atrial fibrillation or flutter**

| **Variables** | **HR** | **95%CI** | **P value** |
| --- | --- | --- | --- |
| **Presenting region** |  |  |  |
| Australian Capital Territory/New South Wales | Reference |  |  |
| Northern Territory/South Australia | 1.00 | 0.97 – 1.03 | 0.968 |
| New Zealand | 1.01 | 0.99 – 1.03 | 0.299 |
| Queensland | 1.06 | 1.04 – 1.08 | <0.001 |
| Tasmania | 0.93 | 0.88 – 0.98 | 0.008 |
| Victoria | 1.01 | 0.99 – 1.03 | 0.232 |
| Western Australia | 1.09 | 1.06 – 1.12 | <0.001 |
|  |  |  |  |
| **Age group** |  |  |  |
| 18-34 years | Reference |  |  |
| 35-49 years | 1.09 | 1.04 – 1.14 | <0.001 |
| 50-64 years | 1.16 | 1.12 – 1.21 | <0.001 |
| 65-79 years | 1.07 | 1.03 – 1.12 | <0.001 |
| ≥80 years | 1.00 | 0.96 – 1.04 | 0.976 |
|  |  |  |  |
| **Sex** |  |  |  |
| Male | Reference |  |  |
| Female | 1.00 | 0.99 – 1.02 | 0.664 |
|  |  |  |  |
| **Hospital sector** |  |  |  |
| Public hospital | Reference |  |  |
| Private hospital | 1.03 | 1.00 – 1.05 | 0.017 |
|  |  |  |  |
| **Procedures performed during the index hospitalisation** |  |  |  |
| Catheter ablation of AF | 0.90 | 0.79 – 1.02 | 0.100 |
| Coronary angiogram/PCI | 1.01 | 0.97 – 1.05 | 0.756 |
| Direct cardioversion | 1.13 | 1.10 – 1.16 | <0.001 |
|  |  |  |  |
| **Cardiovascular comorbidities** |  |  |  |
| Hypertension | 1.03 | 1.00 – 1.06 | 0.095 |
| Congestive heart failure | 0.98 | 0.95 – 1.00 | 0.024 |
| Valvular heart disease | 0.95 | 0.91 – 1.00 | 0.046 |
| Coronary artery disease | 1.03 | 1.00 – 1.06 | 0.090 |
| Vascular disease | 0.98 | 0.93 – 1.03 | 0.465 |
| History of hospitalisation with AF or flutter | 1.12 | 1.09 – 1.14 | <0.001 |
|  |  |  |  |
|  |  |  |  |
| **Non-cardiovascular comorbidities** |  |  |  |
| Diabetes | 0.98 | 0.95 – 1.01 | 0.205 |
| Chronic lung diseases | 0.91 | 0.86 – 0.95 | <0.001 |
| Chronic kidney disease | 0.95 | 0.91 – 0.99 | 0.011 |
| History of stroke | 0.97 | 0.92 – 1.03 | 0.335 |
| Haematological disorders | 0.95 | 0.91 – 0.99 | 0.008 |
| History of pneumonia | 0.94 | 0.89 – 0.98 | 0.010 |
| Musculo-skeletal and connective tissue disorders | 1.01 | 0.98 – 1.04 | 0.420 |
| Major cancer | 0.78 | 0.73 – 0.83 | <0.001 |
| Other cancers | 1.01 | 0.98 – 1.05 | 0.559 |
| Dementia | 0.71 | 0.64 – 0.78 | <0.001 |
| Drug and alcohol-related disorders | 1.02 | 0.96 – 1.09 | 0.478 |
| Mental | 1.07 | 1.01 – 1.14 | 0.025 |
| Skin ulcers | 0.83 | 0.76 – 0.91 | <0.001 |
| Urinary tract infection and incontinence | 0.95 | 0.91 – 0.98 | 0.003 |
| Neurological disorders and paralysis | 0.88 | 0.83 – 0.95 | <0.001 |
| Septicaemia or shock | 1.04 | 0.96 – 1.13 | 0.343 |
| Protein-calorie malnutrition | 0.90 | 0.84 – 0.96 | 0.002 |
| Disorders of fluid or electrolyte or acid-base balance | 0.95 | 0.92 – 0.99 | 0.008 |
| Intestinal obstruction or perforation | 0.99 | 0.89 – 1.10 | 0.839 |
| Delirium and encephalopathy | 0.91 | 0.86 – 0.96 | 0.001 |
| Asthma | 1.09 | 0.99 – 1.21 | 0.094 |
| Pleural effusion or pneumothorax | 0.95 | 0.88 – 1.03 | 0.190 |

Footnote: AF=atrial fibrillation, CI=confidence interval, HR=hazard ratio, sHR=sub-distributional hazard ratio.

**Table S4**: **Variables associated with the outcome of** **re-hospitalisation for catheter ablation of atrial fibrillation**

| **Variables** | **HR** | **95%CI** | **P value** |
| --- | --- | --- | --- |
| **Presenting region** |  |  |  |
| Australian Capital Territory/New South Wales | Reference |  |  |
| Northern Territory/South Australia | 1.12 | 1.03 – 1.22 | 0.010 |
| New Zealand | 0.87 | 0.81 – 0.93 | <0.001 |
| Queensland | 1.03 | 0.97 – 1.09 | 0.313 |
| Tasmania | 0.47 | 0.38 – 0.59 | <0.001 |
| Victoria | 1.02 | 0.96 – 1.08 | 0.503 |
| Western Australia | 1.42 | 1.33 – 1.51 | <0.001 |
|  |  |  |  |
| **Age group** |  |  |  |
| 18-34 years | Reference |  |  |
| 35-49 years | 1.27 | 1.14 – 1.43 | <0.001 |
| 50-64 years | 1.37 | 1.23 – 1.53 | <0.001 |
| 65-79 years | 0.90 | 0.80 – 1.00 | 0.051 |
| ≥80 years | 0.44 | 0.39 – 0.50 | <0.001 |
|  |  |  |  |
| **Sex** |  |  |  |
| Male | Reference |  |  |
| Female | 0.85 | 0.81 – 0.88 | <0.001 |
|  |  |  |  |
| **Hospital sector** |  |  |  |
| Public hospital | Reference |  |  |
| Private hospital | 1.56 | 1.48 – 1.65 | <0.001 |
|  |  |  |  |
| **Procedures performed during the index hospitalisation** |  |  |  |
| Catheter ablation of AF | 1.76 | 1.42 – 2.17 | <0.001 |
| Coronary angiogram/PCI | 1.05 | 0.95 – 1.16 | 0.316 |
| Direct cardioversion | 1.62 | 1.54 – 1.70 | <0.001 |
|  |  |  |  |
| **Cardiovascular comorbidities** |  |  |  |
| Hypertension | 0.93 | 0.85 – 1.02 | 0.125 |
| Congestive heart failure | 0.87 | 0.81 – 0.93 | <0.001 |
| Valvular heart disease | 0.78 | 0.68 – 0.90 | 0.001 |
| Coronary artery disease | 1.02 | 0.93 – 1.11 | 0.730 |
| Vascular disease | 0.92 | 0.79 – 1.08 | 0.330 |
| History of hospitalisation with AF or flutter | 1.30 | 1.22 – 1.38 | <0.001 |
|  |  |  |  |
|  |  |  |  |
| **Non-cardiovascular comorbidities** |  |  |  |
| Diabetes | 0.88 | 0.79 – 0.97 | 0.007 |
| Chronic lung diseases | 0.79 | 0.67 – 0.94 | 0.007 |
| Chronic kidney disease | 0.80 | 0.70 – 0.93 | 0.004 |
| History of stroke | 0.98 | 0.82 – 1.18 | 0.823 |
| Haematological disorders | 0.90 | 0.80 – 1.02 | 0.093 |
| History of pneumonia | 0.92 | 0.79 – 1.08 | 0.311 |
| Musculo-skeletal and connective tissue disorders | 1.09 | 1.00 – 1.19 | 0.040 |
| Major cancer | 0.48 | 0.38 – 0.60 | <0.001 |
| Other cancers | 1.04 | 0.95 – 1.14 | 0.355 |
| Dementia | 0.38 | 0.27 – 0.53 | <0.001 |
| Drug and alcohol-related disorders | 0.78 | 0.64 – 0.96 | 0.017 |
| Mental | 0.98 | 0.81 – 1.17 | 0.809 |
| Skin ulcers | 0.82 | 0.59 – 1.14 | 0.240 |
| Urinary tract infection and incontinence | 0.94 | 0.84 – 1.05 | 0.243 |
| Neurological disorders and paralysis | 0.82 | 0.66 – 1.02 | 0.080 |
| Septicaemia or shock | 1.06 | 0.81 – 1.38 | 0.672 |
| Protein-calorie malnutrition | 0.85 | 0.66 – 1.09 | 0.194 |
| Disorders of fluid or electrolyte or acid-base balance | 0.94 | 0.83 – 1.05 | 0.266 |
| Intestinal obstruction or perforation | 0.89 | 0.65 – 1.21 | 0.439 |
| Delirium and encephalopathy | 0.89 | 0.73 – 1.08 | 0.242 |
| Cardiorespiratory failure and shock | 0.98 | 0.77 – 1.24 | 0.859 |
| Pleural effusion or pneumothorax | 0.87 | 0.68 – 1.12 | 0.282 |
| Cataract | 1.03 | 0.91 – 1.17 | 0.662 |
| Incontinence | 0.78 | 0.55 – 1.10 | 0.153 |
| Cellulitis, local skin infection | 0.85 | 0.69 – 1.04 | 0.118 |

Footnote: AF=atrial fibrillation, CI=confidence interval, HR=hazard ratio, sHR=sub-distributional hazard ratio.

**Table S5: Baseline characteristics by survival status at the end of the study period**

|  | **All-cause mortality** | |
| --- | --- | --- |
|  | **Survived**  **(N=191,325), n (%)** | **Died**  **(N=69,167), n (%)** |
| **Patients’ demographics** |  |  |
| Age (mean ± SD) | 67.1±14.2 | 79.7±10.3 |
| Age group |  |  |
| 18-34 | 5,094 (2.7) | 117 (0.2) |
| 35-49 | 17,482 (9.1) | 825 (1.2) |
| 50-64 | 51,974 (27.2) | 5,066 (7.3) |
| 65-79 | 79,231 (41.4) | 23,091 (33.4) |
| ≥80 | 37,544 (19.6) | 40,068 (57.9) |
| Female (%) | 91,553 (47.9) | 37,742 (54.6) |
| Median length of stay (IQR) | 2 (1 – 4) | 3 (1 – 7) |
| Geographical region |  |  |
| New Zealand | 29,259 (15.3) | 12,356 (17.9) |
| Australian Capital Territory/  New South Wales | 56,603 (29.6) | 21,058 (30.5) |
| South Australia/Northern Territory | 12,213 (6.4) | 4,607 (6.7) |
| Queensland | 36,678 (19.2) | 11,695 (16.9) |
| Tasmania | 2,756 (1.4) | 1,166 (1.7) |
| Victoria | 39,646 (20.7) | 13,442 (19.4) |
| Western Australia | 14,170 (7.4) | 4,843 (7.0) |
| Treatment at a Private hospital | 24,227 (12.7) | 7,577 (11.0) |
|  |  |  |
| **Procedures during the index hospitalisation** |  |  |
| Catheter ablation | 748 (0.4) | 112 (0.2) |
| Cardioversion | 20,234 (10.6) | 3,211 (4.6) |
| Coronary angiogram | 6,310 (3.3) | 1,305 (1.9) |
| PCI | 418 (0.2) | 136 (0.2) |
|  |  |  |
| **CHA_2_DS_2_-VASc score^a^** | 1 (0 – 2) | 2 (2 – 3) |
|  |  |  |
| **Cardiovascular History** |  |  |
| Hypertension | 35,644 (18.6) | 22,786 (32.9) |
| Heart failure | 24,798 (13.0) | 21,275 (30.8) |
| Valvular and rheumatic heart disease | 8,544 (4.5) | 4,995 (7.2) |
| Coronary artery disease | 18,915 (9.9) | 11,672 (16.9) |
| Vascular disease | 3,104 (1.6) | 2,756 (4.0) |
| History of AF or flutter^b^ | 20,546 (10.7) | 14,062 (20.3) |
|  |  |  |
| **Other Comorbidities** |  |  |
| Diabetes mellitus | 25,128 (13.1) | 12,408 (17.9) |
| Chronic lung diseases | 5,849 (3.1) | 7,463 (10.8) |
| Chronic kidney disease | 6,071 (3.2) | 7,432 (10.8) |
| Previous stroke or TIA | 2,947 (1.5) | 2,196 (3.2) |
| Haematological disorders | 9,466 (5.0) | 9,764 (14.1) |
| Pneumonia | 6,757 (3.5) | 6,911 (10.0) |
| Musculo-skeletal and connective tissue disorders | 15,123 (7.9) | 9,658 (14.0) |
| Dementia and senility | 1,689 (0.9) | 4,392 (6.4) |
| Major cancer | 2,019 (1.1) | 4,946 (7.2) |
| End-stage liver disease | 502 (0.3) | 546 (0.8) |
| Drug or alcohol abuse, psychosis or dependence | 6,468 (3.4) | 2,799 (4.1) |
| Psychiatric disorders | 3,110 (4.5) | 4,228 (2.2) |
| Neurological disorders and paralysis | 2,429 (1.3) | 2,777 (4.0) |
| Skin ulcers | 785 (0.4) | 1,725 (2.5) |
| Urinary tract disorders and incontinence | 7,823 (4.1) | 7,584 (11.0) |

Footnote: SD=standard deviation, IQR=interquartile range, PCI=percutaneous coronary intervention, TIA=transient ischemic attack. ^a^CHA_2_DS_2_-VASc score is a score used to evaluate risk of experiencing thromboembolic events of AF patients in which a point each is given for the presence of congestive heart failure (C), hypertension (H), age >=65 years old (A), diabetes (D), vascular disease (VASc) and female gender and 2 points each are given for age>=75 years old and history of stroke (S). The total score ranges from 0 to 9 with the higher the score, the higher the risk (1). ^b^History of AF or flutter was derived from all acute and elective hospital encounters in the preceding 12 months.

**Table S6:** **Baseline characteristics by sex**

|  | **Overall**  **(N=260,492), n (%)** | **Male**  **(N=131,197), n(%)** | **Female**  **(N=129,295), n(%)** | **P value** |
| --- | --- | --- | --- | --- |
| **Patients’ demographics** |  |  |  |  |
| **Age (mean ± SD)** | 70.5±14.4 | 66.8±14.8 | 74.2±12.8 | <0.001 |
| **Age group** |  |  |  |  |
| 18-34 | 5,211 (2.0) | 4,144 (3.2) | 1,067 (0.8) | <0.001 |
| 35-49 | 18,307 (7.0) | 13,352 (10.2) | 4,955 (3.8) |  |
| 50-64 | 57,040 (21.9) | 35,692 (27.2) | 21,348 (16.5) |  |
| 65-79 | 102,322 (39.3) | 50,760 (38.7) | 51,562 (39.9) |  |
| ≥80 | 77,612 (29.8) | 27,249 (20.8) | 50,363 (39.0) |  |
|  |  |  |  |  |
| **Median length of stay (IQR)** | 2 (1 – 5) | 2 (1 – 4) | 2 (1 – 5) | <0.001 |
| **Geographical region** |  |  |  |  |
| New Zealand | 41,615 (16.0) | 21,033 (16.1) | 20,582 (15.9) |  |
| Australian Capital Territory/  New South Wales | 77,661 (29.8) | 39,135 (29.8) | 38,526 (29.8) |  |
| South Australia/Northern Territory | 16,820 (6.5) | 8,433 (6.4) | 8,387 (6.5) | <0.001 |
| Queensland | 48,373 (18.6) | 24,904 (19.0) | 23,469 (18.2) |  |
| Tasmania | 3,922 (1.5) | 1,991 (1.5) | 1,931 (1.5) |  |
| Victoria | 53,088 (20.4) | 25,867 (19.7) | 27,221 (21.1) |  |
| Western Australia | 19,013 (7.3) | 9,834 (7.5) | 9,179 (7.1) |  |
|  |  |  |  |  |
| **Treatment at a Private hospital** | 31,804 (12.2) | 15,584 (11.9) | 16,220 (12.5) | <0.001 |
|  |  |  |  |  |
| **Procedures performed during the index hospitalisation** | | | | |
| Catheter ablation | 860 (0.3) | 623 (0.5) | 237 (0.2) | <0.001 |
| Cardioversion | 23,445 (9.0) | 15,480 (11.8) | 7,965 (6.2) | <0.001 |
| Coronary angiogram | 7,615 (2.9) | 4,608 (3.5) | 3,007 (2.3) | <0.001 |
| PCI | 554 (0.2) | 374 (0.3) | 180 (0.2) | <0.001 |
|  |  |  |  |  |
| **CHA_2_DS_2_-VASc score^a^** | 2 (1 – 3) | 1 (0 – 2) | 2 (1 – 3) | <0.001 |
|  |  |  |  |  |
| **Cardiovascular History** |  |  |  |  |
| Hypertension | 58,430 (22.4) | 27,659 (21.1) | 31,074 (24.0) | <0.001 |
| Heart failure | 46,073 (17.7) | 22,237 (17.0) | 23,836 (18.5) | <0.001 |
| Valvular and rheumatic heart disease | 13,539 (5.2) | 6,586 (5.0) | 6,953 (5.4) | <0.001 |
| Coronary artery disease | 30,587 (11.7) | 17,647 (13.5) | 12,940 (10.0) | <0.001 |
| Vascular disease | 5,860 (2.3) | 3,105 (2.4) | 2,755 (2.1) | <0.001 |
| History of AF or flutter^b^ | 34,608 (13.3) | 17,320 (13.2) | 17,288 (13.4) | 0.203 |
|  |  |  |  |  |
| **Other Comorbidities** |  |  |  |  |
| Diabetes mellitus | 37,536 (14.4) | 19,762 (15.1) | 17,774 (13.8) | <0.001 |
| Chronic lung diseases | 13,312 (5.1) | 7,294 (5.6) | 6,018 (4.7) | <0.001 |
| Chronic kidney disease | 13,503 (5.2) | 7,166 (5.5) | 6,337 (4.9) | <0.001 |
| Previous stroke or TIA | 5,143 (2.0) | 2,335 (1.8) | 2,808 (2.2) | <0.001 |
| Haematological disorders | 19,230 (7.4) | 9,175 (7.0) | 10,055 (7.8) | <0.001 |
| Pneumonia | 13,668 (5.3) | 7,074 (5.4) | 6,594 (5.1) | 0.001 |
| Musculo-skeletal and connective tissue disorders | 24,781 (9.5) | 11,151 (8.5) | 13,630 (10.5) | <0.001 |
| Dementia and senility | 6,081 (2.3) | 2,267 (1.7) | 3,814 (3.0) | <0.001 |
| Major cancer | 6,965 (2.7) | 4,119 (3.1) | 2,846 (2.2) | <0.001 |
| End-stage liver disease | 1,047 (0.4) | 722 (0.6) | 326 (0.3) | <0.001 |
| Drug or alcohol abuse, psychosis or dependence | 9,267 (3.6) | 7,013 (5.4) | 2,254 (1.7) | <0.001 |
| Psychiatric disorders | 7,338 (2.8) | 3,291 (2.5) | 4,047 (3.1) | <0.001 |
| Neurological disorders and paralysis | 5,206 (2.0) | 2,524 (1.9) | 2,682 (2.1) | 0.006 |
| Skin ulcers | 2,510 (1.0) | 1,087 (0.8) | 1,423 (1.1) | <0.001 |
| Urinary tract disorders and incontinence | 15,407 (5.9) | 7,352 (5.6) | 8,055 (6.2) | <0.001 |

Footnote: SD=standard deviation, IQR=interquartile range, PCI=percutaneous coronary intervention, TIA=transient ischemic attack. ^a^CHA_2_DS_2_-VASc score is a score used to evaluate risk of experiencing thromboembolic events of AF patients in which a point each is given for the presence of congestive heart failure (C), hypertension (H), age >=65 years old (A), diabetes (D), vascular disease (VASc) and female gender and 2 points each are given for age>=75 years old and history of stroke (S). The total score ranges from 0 to 9 with the higher the score, the higher the risk (1). ^b^History of AF or flutter was derived from all acute and elective hospital encounters in the preceding 12 months.

**Table S7:** **Baseline characteristics by two-year enrolment period**

|  | **2008-2009**  **(N=55,746), n (%)** | **2010-2011**  **(N=52,325), n (%)** | **2012-2013**  **(N=50,505), n (%)** | **2014-2015**  **(N=50,090), n (%)** | **2016-2017**  **(N=51,826), n (%)** |
| --- | --- | --- | --- | --- | --- |
| **Patients’ demographics** |  |  |  |  |  |
| Age (mean ± SD) | 69.8±14.5 | 70.2±14.5 | 70.6±14.3 | 70.7±14.3 | 71.0±14.2 |
| Age group |  |  |  |  |  |
| 18-34 | 1,179 (2.1) | 1,132 (2.2) | 963 (1.9) | 966 (1.9) | 971 (1.9) |
| 35-49 | 4,310 (7.7) | 3,879 (7.4) | 3,430 (6.8) | 3,391 (6.8) | 3,297 (6.4) |
| 50-64 | 12,784 (22.9) | 11,641 (22.3) | 10,950 (21.7) | 10,711 (21.4) | 10,954 (21.1) |
| 65-79 | 21,641 (38.8) | 20,070 (38.4) | 19,903 (39.4) | 19,841 (39.6) | 20,867 (40.3) |
| ≥80 | 15,832 (28.4) | 15,603 (29.8) | 15,259 (30.2) | 15,181 (30.3) | 15,737 (30.4) |
|  |  |  |  |  |  |
| Female | 20,867 (50.4) | 26,093 (49.9) | 24,905 (49.3) | 24,667 (49.3) | 25,563 (49.3) |
|  |  |  |  |  |  |
| Median length of stay (IQR) | 2 (1 – 5) | 2 (1 – 5) | 2 (1 – 5) | 2 (1 – 4) | 2 (1 – 4) |
| Geographical region |  |  |  |  |  |
| New Zealand | 9,583 (17.2) | 8,600 (16.4) | 8,049 (15.9) | 7,794 (15.6) | 7,589 (14.6) |
| Australian Capital Territory/  New South Wales | 16,642 (29.9) | 15,520 (29.7) | 15,278 (30.3) | 15,002 (30.0) | 15,219 (29.4) |
| South Australia/Northern Territory | 3,506 (6.3) | 3,458 (6.6) | 3,267 (6.5) | 3,287 (6.6) | 3,302 (6.4) |
| Queensland | 9,556 (17.1) | 9,032 (17.3) | 9,599 (19.0) | 9,977 (19.9) | 10,209 (19.7) |
| Tasmania | 945 (1.7) | 660 (1.3) | 668 (1.3) | 766 (1.5) | 893 (1.7) |
| Victoria | 11,698 (21.0) | 11,022 (21.1) | 9,746 (19.3) | 9,768 (19.5) | 10,854 (20.9) |
| Western Australia | 3,816 (6.9) | 4,033 (7.7) | 3,898 (7.7) | 3,506 (7.0) | 3,760 (7.3) |
|  |  |  |  |  |  |
| Treatment at a Private hospital | 6,623 (11.9) | 6,247 (11.9) | 5,994 (11.9) | 6,084 (12.2) | 6,856 (13.2) |
|  |  |  |  |  |  |
| **Procedures performed during the index hospitalisation** | | | | | |
| Catheter ablation | 118 (0.2) | 162 (0.3) | 183 (0.4) | 197 (0.4) | 200 (0.4) |
| Cardioversion | 4,698 (8.4) | 4,294 (8.2) | 4,532 (9.0) | 4,710 (9.4) | 5,211 (10.1) |
| Coronary angiogram | 1,237 (2.2) | 1,414 (2.7) | 1,550 (3.1) | 1,617 (3.2) | 1,797 (3.5) |
| PCI | 94 (0.2) | 99 (0.2) | 108 (0.2) | 112 (0.2) | 141 (0.3) |
|  |  |  |  |  |  |
| **CHA_2_DS_2_-VASc score^a^** | 2 (1 – 3) | 2 (1 – 2) | 2 (1 – 3) | 2 (1 – 3) | 2 (1 – 3) |
|  |  |  |  |  |  |
| **Cardiovascular History** |  |  |  |  |  |
| Hypertension | 16,792 (30.0) | 11,897 (22.7) | 11,692 (23.2) | 10,571 (21.1) | 7,871 (15.2) |
| Heart failure | 9,738 (17.5) | 8,938 (17.2) | 8,963 (17.8) | 8,899 (17.8) | 9,490 (18.3) |
| Valvular and rheumatic heart disease | 3,238 (5.8) | 2,554 (4.9) | 2,694 (5.3) | 2,536 (5.1) | 2,517 (4.9) |
| Coronary artery disease | 8,140 (14.6) | 6,280 (12.0) | 5,774 (11.3) | 5,153 (10.3) | 5,240 (10.1) |
| Vascular disease | 1,277 (2.3) | 1,138 (2.2) | 1,193 (2.4) | 1,083 (2.2) | 1,169 (2.3) |
| History of AF or flutter hospitalization^b^ | 9,086 (16.3) | 6,624 (12.3) | 6,255 (12.4) | 6,242 (12.5) | 6,401 (12.4) |
|  |  |  |  |  |  |
| **Other Comorbidities** |  |  |  |  |  |
| Diabetes mellitus | 6,546 (11.7) | 3,677 (7.0) | 7,667 (15.2) | 9,527 (19.0) | 10,119 (19.5) |
| Chronic lung diseases | 2,878 (5.2) | 2,466 (4.7) | 2,590 (5.1) | 2,632 (5.3) | 2,746 (5.3) |
| Chronic kidney disease | 2,408 (4.3) | 2,124 (4.1) | 2,556 (5.1) | 3,085 (6.2) | 3,330 (6.4) |
| Previous stroke or TIA | 1,140 (2.0) | 1,017 (1.9) | 1,024 (2.0) | 975 (2.0) | 987 (1.9) |
| Haematological disorders | 3,929 (7.1) | 3,650 (7.0) | 3,695 (7.3) | 3,858 (7.7) | 4,098 (7.9) |
| Pneumonia | 2,468 (4.4) | 2,515 (4.8) | 2,633 (5.2) | 2,885 (5.8) | 3,167 (6.1) |
| Musculo-skeletal and connective tissue disorders | 5,004 (9.0) | 4,780 (9.2) | 4,810 (9.5) | 4,922 (9.8) | 5,265 (10.2) |
| Dementia and senility | 1,439 (2.6) | 1,208 (2.3) | 1,173 (2.3) | 1,186 (2.4) | 1,075 (2.1) |
| Major cancer | 1,306 (2.3) | 1,318 (2.5) | 1,342 (2.7) | 1,479 (3.0) | 1,520 (2.9) |
| End-stage liver disease | 208 (0.4) | 166 (0.3) | 194 (0.4) | 229 (0.5) | 251 (0.5) |
| Drug or alcohol abuse, psychosis or dependence | 1,545 (2.8) | 1,421 (2.7) | 1,722 (3.4) | 2,050 (4.1) | 2,529 (4.9) |
| Psychiatric disorders | 1,542 (2.8) | 1,450 (2.8) | 1,409 (2.8) | 1,437 (2.9) | 1,500 (2.9) |
| Neurological disorders and paralysis | 1,076 (1.9) | 971 (1.9) | 1,008 (2.0) | 1,059 (2.1) | 1,092 (2.1) |
| Skin ulcers | 558 (1.0) | 562 (1.1) | 551 (1.1) | 578 (1.2) | 261 (0.5) |
| Urinary tract disorders and incontinence | 3,147 (5.7) | 2,924 (5.6) | 3,043 (6.0) | 3,110 (6.2) | 3,183 (6.1) |

Footnote: SD=standard deviation, IQR=interquartile range, PCI=percutaneous coronary intervention, TIA=transient ischemic attack. ^a^CHA_2_DS_2_-VASc score is a score used to evaluate risk of experiencing thromboembolic events of AF patients in which a point each is given for the presence of congestive heart failure (C), hypertension (H), age >=65 years old (A), diabetes (D), vascular disease (VASc) and female gender and 2 points each are given for age>=75 years old and history of stroke (S). The total score ranges from 0 to 9 with the higher the score, the higher the risk (1). ^b^History of AF or flutter was derived from all acute and elective hospital encounters in the preceding 12 months.

**References**

1. Hindricks G, Potpara T, Dagres N, Arbelo E, Bax JJ, Blomstrom-Lundqvist C, et al. 2020 ESC Guidelines for the diagnosis and management of atrial fibrillation developed in collaboration with the European Association for Cardio-Thoracic Surgery (EACTS): The Task Force for the diagnosis and management of atrial fibrillation of the European Society of Cardiology (ESC) Developed with the special contribution of the European Heart Rhythm Association (EHRA) of the ESC. Eur Heart J. 2021;42(5):373-498.
